# Resolutions of Respect Adopted by the Dentists of Nashville to the Late Dr. Hamlin

**Published:** 1874-08

**Authors:** 


### RESOLUTIONS OF RESPECT ADOPTED BY THE DENTISTS OF NASHVILLE TO THE LATE DR. HAMLIN.

At a meeting of the Dentists of Nashville, held recently, at the office of Dr. Ross for the purpose of paying a last tribute of respect to the memory of Dr. T. B. Hamlin, the following proceedings were had:

Dr. Ross was appointed Chairman of the meeting and Dr. Noel Secretary.

A committee of three composed of Drs. Morgan, King and Cobb, were appointed to draft suitable resolutions. The committee presented the following which were adopted:

*Whereas*, God in his providence has taken to himself our highly esteemed friend and former co-laborer, Theodore Burnam Hamlin, D. D. S., and believing the Almighty Father doeth all things well, and that his providences are always wise and good, we bow with humble submission to his Divine will.

*Resolved*, that we here record our high appreciation of his character, both as a Christian gentleman and a professional man. And we would call to mind his marked energy of character, his concentration of purpose, and his lofty aspirations for perfection as worthy of imitation.

*Resolved*, that we cherish the memory of his life, and especially of his professional life, and hold it up as worthy of imitation by our professional brethren.

*Resolved*, that in his death, his family lose a kind and affectionate husband and father, his church an upright, zealous member and the Masonic fraternity one of its brightest jewels

*Resolved*, that we will attend his funeral services in a body.

*Resolved*, that a copy of these proceedings be sent to our daily papers for publication, and that a copy be furnished the family of the deceased.

*Resolved*, that a copy of these resolutions be furnished all the dental journals for publication.

